# A review on the role of MiR-193a-5p in oncogenesis and tumor progression

**DOI:** 10.3389/fonc.2025.1543215

**Published:** 2025-03-14

**Authors:** Weixiang Tang, Yuhua Rao, Longsheng Pi, Jinping Li

**Affiliations:** Department of Orthopaedics, Changsha Central Hospital (The Affiliated Changsha Central Hospital, Hengyang Medical School, University of South China), Changsha, Hunan, China

**Keywords:** miR-193a-5p, cancer, expression, malignancies, modulation, oncogenesis

## Abstract

MicroRNA (miRNA), a class of short non-coding RNA molecules comprising 18-25 nucleotides, are pivotal regulators of gene expression within physiological environments, influencing processes such as cell growth, apoptosis, proliferation, differentiation, migration (including cellular movement), and angiogenesis. They also play a crucial role in disease progression, invasion, and metastasis. Specifically, miR-193a-5p, a member of the miR-193a family, is instrumental in the development of various malignancies, including osteosarcoma, hepatocellular carcinoma, cervical cancer, melanoma, gastrointestinal cancer, lung cancer, prostate cancer, and bladder cancer. Studies have revealed that miR-193a-5p (sequence: UGGGUCUUUGCGGGCGAGAUGA; accession number: MIMAT0004614) is downregulated in numerous cancer cell lines and clinical samples. Furthermore, the tumor-suppressive effects of miR-193a-5p have been corroborated in animal models across different cancer types. These studies suggest that overexpression of this miRNA or modulation of lncRNA expression can inhibit oncogenesis. In this review, we summarize the functions of miR-193a-5p in cancer development.

## Introduction

MicroRNAs (miRNAs) are small transcripts that regulate gene expression at the post-transcriptional level by specifically targeting mRNA. These miRNAs, approximately 18-25 nucleotides in length, originate from the coding and non-coding transcriptional units of introns, exons, or intergenic regions (1). They are produced through a multistep process involving both nuclear and cytoplasmic proteins. miRNAs are implicated in oncogenic processes as they can regulate the expression of several oncogenes and tumor suppressor genes, as well as activate cancer-related pathways (2). Various miRNA expression patterns and functions have been evaluated across different cancer types. Due to their stability in circulating or other biological fluids, miRNAs represent potential biomarkers for diagnostic and follow-up purposes (3). Dysregulation of miRNAs is associated with cancer progression, making them a valuable molecular tool for the noninvasive assessment of cancer occurrence and prognosis (4).

MiR-193a-5p exemplifies a transcript with a significant role in the development of various malignancies, including adult prostate tumors, osteosarcoma, hepatocellular carcinoma, cervical cancer, gastrointestinal cancer, pancreatic cancer, and nasopharyngeal carcinoma. Multiple studies have investigated the role of miR-193a-5p in carcinogenesis using both *in vitro* and *in vivo* techniques. The expression pattern of miR-193a-5p has also been evaluated in clinical samples from patients with various malignancies. This review summarizes the functions of miR-193a-5p in cancer development based on these lines of evidence (5). The choice of miR-193a-5p for this review is based on its important role in suppressing carcinogenesis, its downregulation in various solid and hematological malignancies, and its potential as an anticancer target. Papers were selected based on the following criteria: publication in full-text English in peer-reviewed journals, and detailed descriptions of the methods used. Additionally, papers had to include *in vitro* functional studies or expression determinations in clinical samples (5).

## Cell line studies

**Figure 1:**
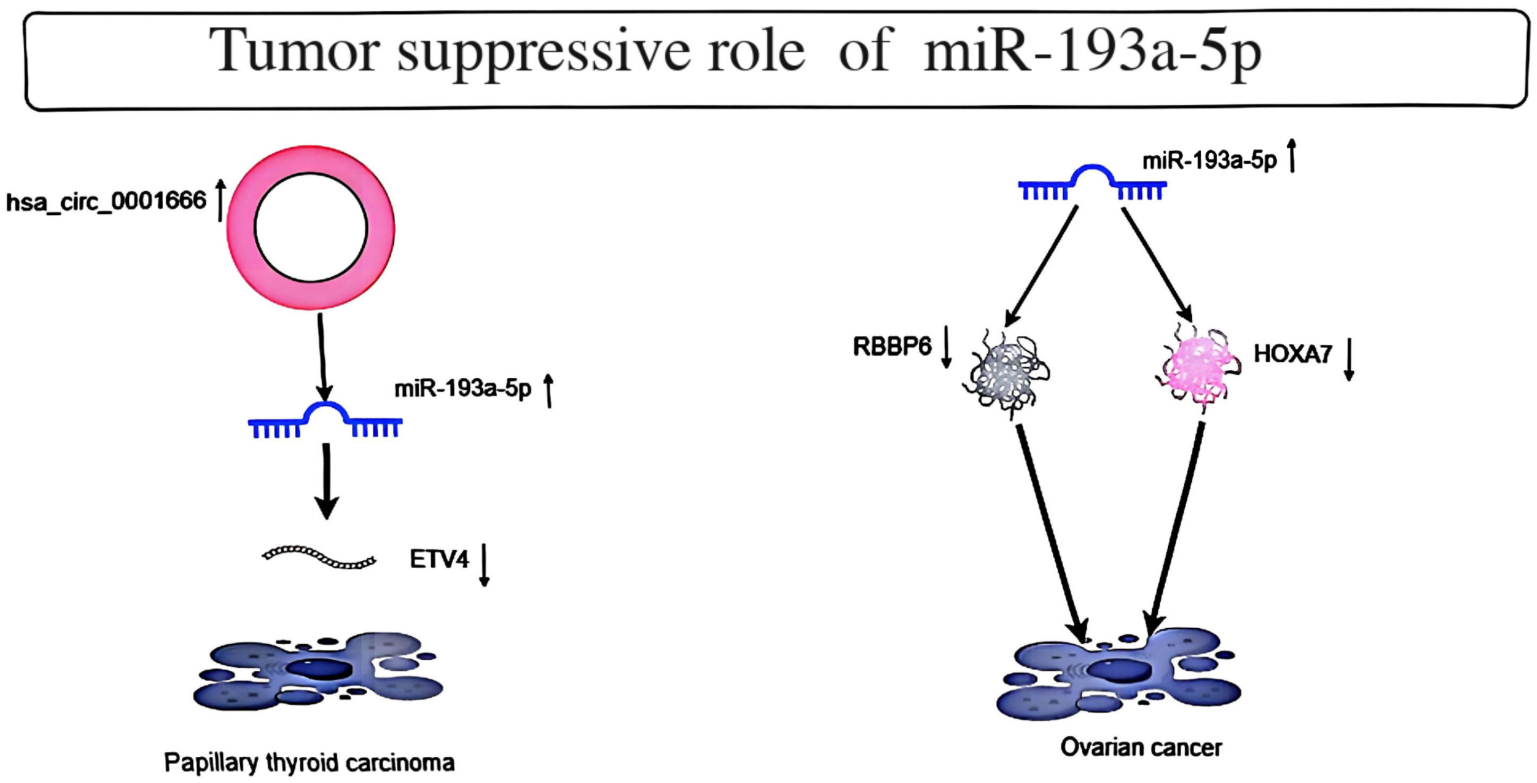
Depicts the tumor suppressor role of MiR-193a-5p in Papillary thyroid carcinoma and Ovarian cancer.

Cell line studies suggest a critical role for miR-193a-5p in oncogenesis. These studies demonstrate that this miRNA promotes the transcription of several genes, particularly known oncogenes. An *in vitro* study on thyroid cancer revealed interactions between miR-330-5p, miR-193a-5p, miR-326, and circular RNA hsa_circ_0001666. According to luciferase reporter turnover, RIP assays, and flow cytometry results, hsa_circ_0001666 shares a common binding motif with ETV4 on miR-330-5p, miR-193a-5p, and miR-326. The downregulation of hsa_circ_0001666 directly regulates the miR-330-5p/miR-193a-5p/miR-326 axis to upregulate the expression of ETV4. The downregulation of hsa_circ_0001666 causes cell cycle arrest in the G1 phase, leading to increased expression levels of pro-apoptotic proteins (including cleaved caspase 3 and caspase 9). Additionally, it also results in the downregulation of ETV4, and the downregulation of ETV4 further promotes the increase in apoptosis rate (6). These findings suggest that circular RNA hsa_circ_0001666 promotes thyroid tumorigenesis through miRNA pathways. Another study in ovarian cancer indicated that upregulation of miR-193a-5p expression may inhibit EOC by promoting SKOV3 cell apoptosis and inhibiting cell proliferation and migration (7). Additionally, miR-193a-5p inhibited HOXA7 expression and induced apoptosis by binding the 3’-untranslated region of HOXA7 mRNA (8). The mechanisms by which miR-193a-5p inhibit certain tumors are briefly demonstrated (**Figure 1**).

**Figure 2:**
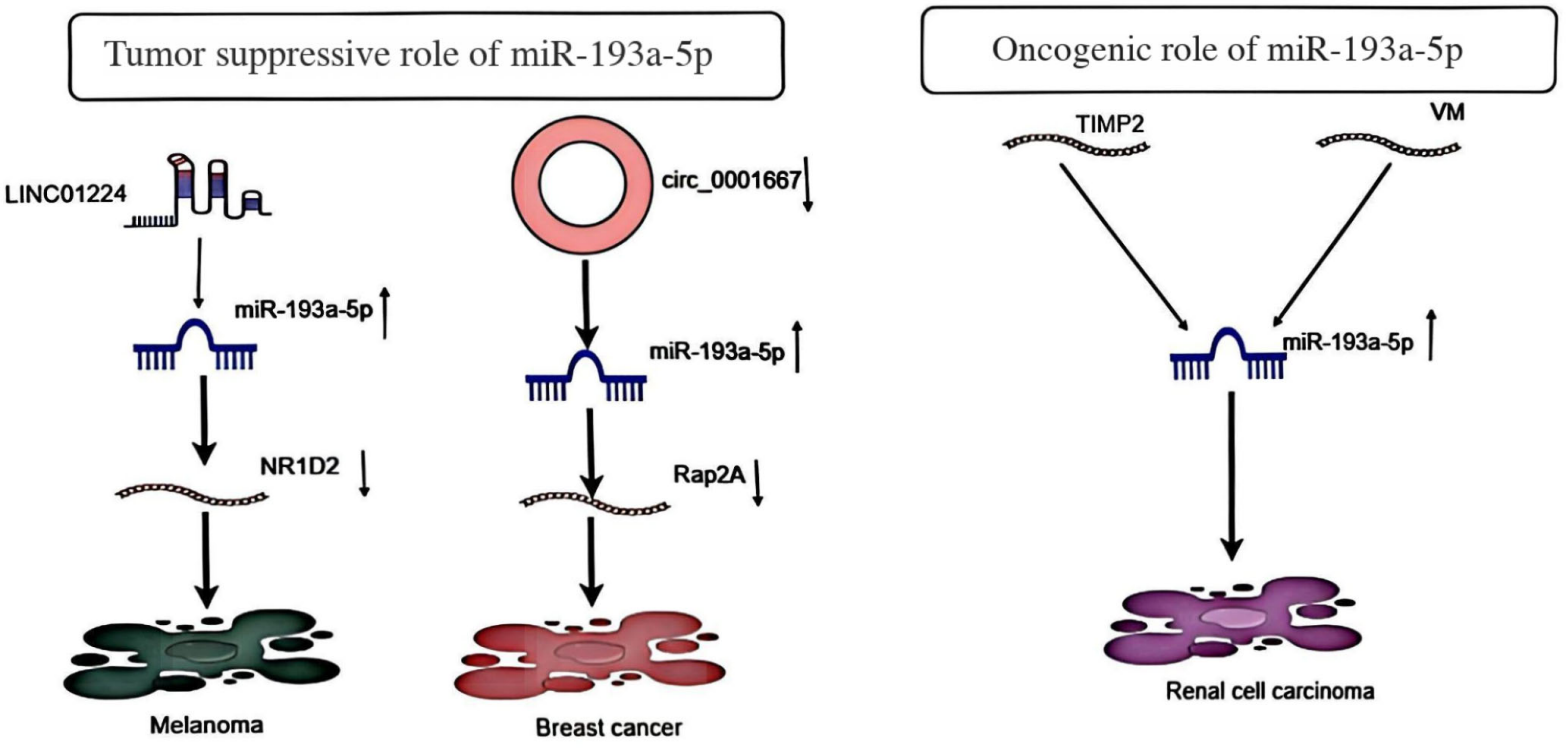
Shows the tumor suppressor impact of MiR-193a-5p in Melanoma and Breast cancer and its oncogenic role in Renal cell carcinoma.

Furthermore, LINC01224 promotes melanoma cell proliferation via the miR-193a-5p/NR1D2 axis and reduces radiosensitivity (9). It has also been demonstrated that circ_0001667, through miR-193a-5p, promotes doxorubicin resistance and tumor development in breast cancer (10). In RCC, studies found that tumor-associated macrophages transfer miR-193a-5p to RCC cells via exosomes, promoting cellular angiogenic mimicry and invasion, ultimately facilitating RCC metastasis. Further investigation revealed that miR-193a-5p is transcriptionally regulated by HIF-1 α, directly targeting the TIMP2 3’-untranslated region (UTR) and thereby downregulating its expression (11). The roles played by miR-193a-5p in different tumors may not be the same (**Figure 2**).

**Figure 3:**
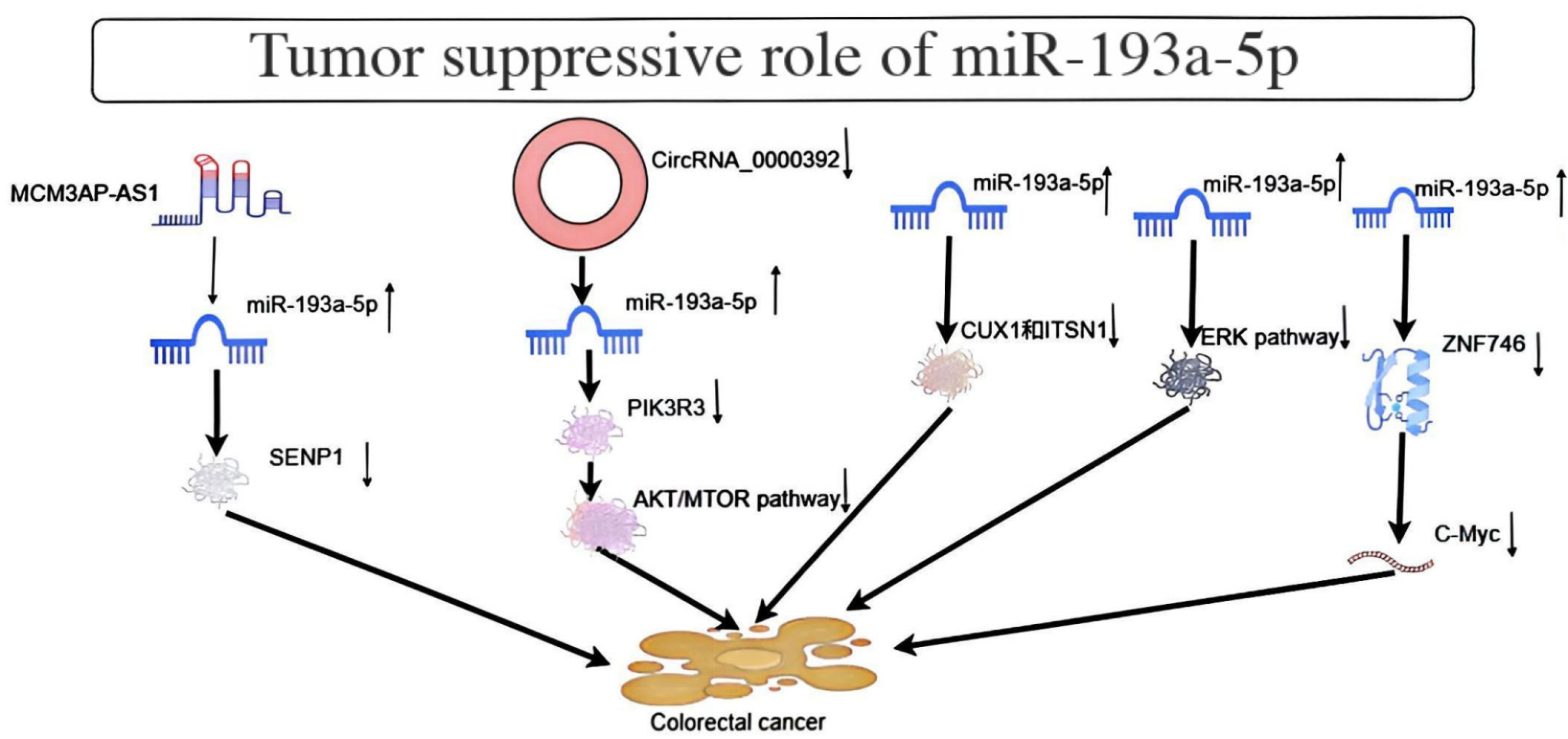
Depicts the tumor suppressor role of MiR-193a-5p in Colorectal cancer.

In colorectal cancer cells, the non-coding RNA (circRNA) circRNA_0000392, targeting miR-193a-5p, was shown to be upregulated. This circRNA acts as an adsorption point for miR-193a-5p, promoting the proliferation, migration, and invasiveness of these cells. PIK3R3 was identified as a target gene of miR-193a-5p in colorectal cancer cells, mediating the effects of miR-193a-5p and circRNA_0000392, and enhancing AKT signaling, the miR-193a-5p inhibitor can alleviate the reduction in PIK3R3 expression caused by circRNA_0000392 siRNA. Downregulating circRNA_0000392 decreases the levels of PIK3R3 protein and the phosphorylation levels of AKT and mTOR, thereby reducing the growth of colorectal cancer cells *in vivo* (12). Another study confirmed the downregulation of miR-193a-5p in colorectal cancer cells (**Figure 3**). Dual luciferase reporter assay and bioinformatics analysis verified the regulatory effect of miR-193a-5p on the expression of homeobox 1 (CUX1) and intersectin 1 (ITSN1). Knockdown of CUX1 and ITSN1 reduced the inhibitory effect of miR-193a-5p on the proliferation and migration of colorectal cancer cells (13). Additionally, miR-193a-5p has been shown to inhibit colorectal cancer cell proliferation and migration via the extracellular signal-regulated kinase (ERK) signaling pathway. It has also been shown that miR-193a-5p is downregulated in colorectal cancer cells, which is associated with early tumorigenesis and advanced lymphatic metastasis (14–17). Moreover, SENP1 was identified as a novel target of miR-193a-5p, and its upregulation by MCM3AP-AS1, which adsorbs miR-193a-5p and inhibits its activity, promotes tumor proliferation, migration, and invasion (18). Finally, another study showed that morusin inhibits the growth of colon cancer cells by inhibiting the expression of c-Myc and zinc finger protein 746 (ZNF746) in HCT116 cells, interfering with the binding of c-Myc and ZNF746, and upregulating miR-193a-5p (19).

**Figure 4:**
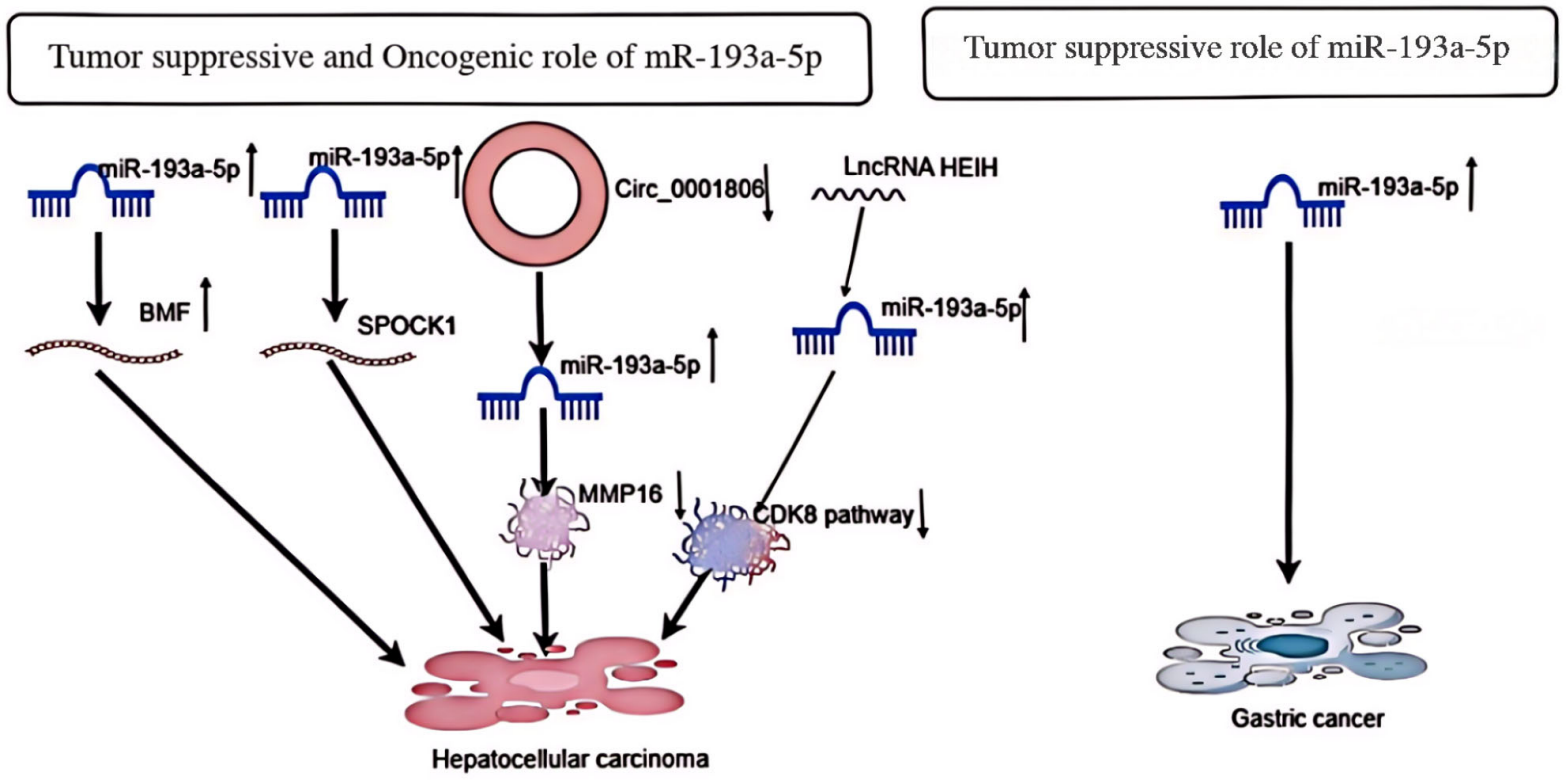
Depicts the oncogenic and tumor suppressive effects of miR-193a-5p in Hcancer and its tumor suppressive effects in gastric cancer.

In HCC cells, overexpression of miR-193a-5p has been associated with increased cell migration and proliferation, induction of cell cycle progression, and promotion of apoptosis. Overexpression of miR-193a-5p can accelerate HCC cell proliferation, promote G1/S transition, and inhibit apoptosis (20). Furthermore, miR-193a-5p can inhibit the proliferation and invasion of HCC cells and promote apoptosis through overexpression (21). According to the first two studies on liver cancer cells, miR-193a-5p exhibits contradictory observations in the tissues of liver cancer patients. It has been reported that miR-193a-5p, which targets BMF to regulate cell proliferation, G1/S transition, and apoptosis, appears to have higher expression in tumors than in non-neoplastic tissues of HCC patients. However, miR-193a-5p targeting SPOCK1 is downregulated in HCC patients (22). Further studies revealed that long non-coding RNA (lncRNA) in silenced hepatocellular carcinoma (HEIH) could bind to miR-193a-5p to promote the expression of cyclin-dependent kinase 8 (CDK 8) in hepatocellular carcinoma (HCC) cells. This mechanism inhibited the proliferation, migration, and invasion of HCC cells through the miR-193a-5p/CDK 8 axis, showing a complex role of miR-193a-5p in the regulation of HCC progression (23). Finally, a study suggested that upregulation of circ _ 0001806 could promote the malignant biological behavior of hepatocellular carcinoma (HCC) by regulating MMP 16 expression by inhibiting miR-193a-5p. This finding further confirms the key role of miR-193a-5p in the development of liver cancer and suggests its possibility as a potential therapeutic target (24). In conclusion, the effects of miR-193a-5p in HCC cells have complex and diverse mechanisms that can both promote and inhibit the development of HCC (**Figure 4**). A deeper understanding of these mechanisms will facilitate the development of more effective treatments for HCC.

An experimental study on gastric cancer cells demonstrated the effect of enhanced miR-193a-5p expression on the proliferation, apoptosis, and migration of gastric cancer cells (**Figure 4**). Enhanced expression of miR-193a-5p, as detected by RT-PCR, had no significant effect on cell survival or apoptosis in transfected cells. Furthermore, this inhibitory function of miR-193a-5p on the mobility of KATO III cell lines coincided with the inhibition of vimentin and MMP-9 gene expression (25). Another study showed that miR-193a-5p expression was significantly reduced in gastric cancer cells compared with adjacent normal tissues, inhibiting the growth of gastric cancer cells (26).This suggests that this tiny RNA has an important role in inhibiting the growth of gastric cancer cells, and these findings not only deepen our understanding of GC pathogenesis, but also provide potential targets for the development of novel therapeutic strategies in the future.

**Figure 5:**
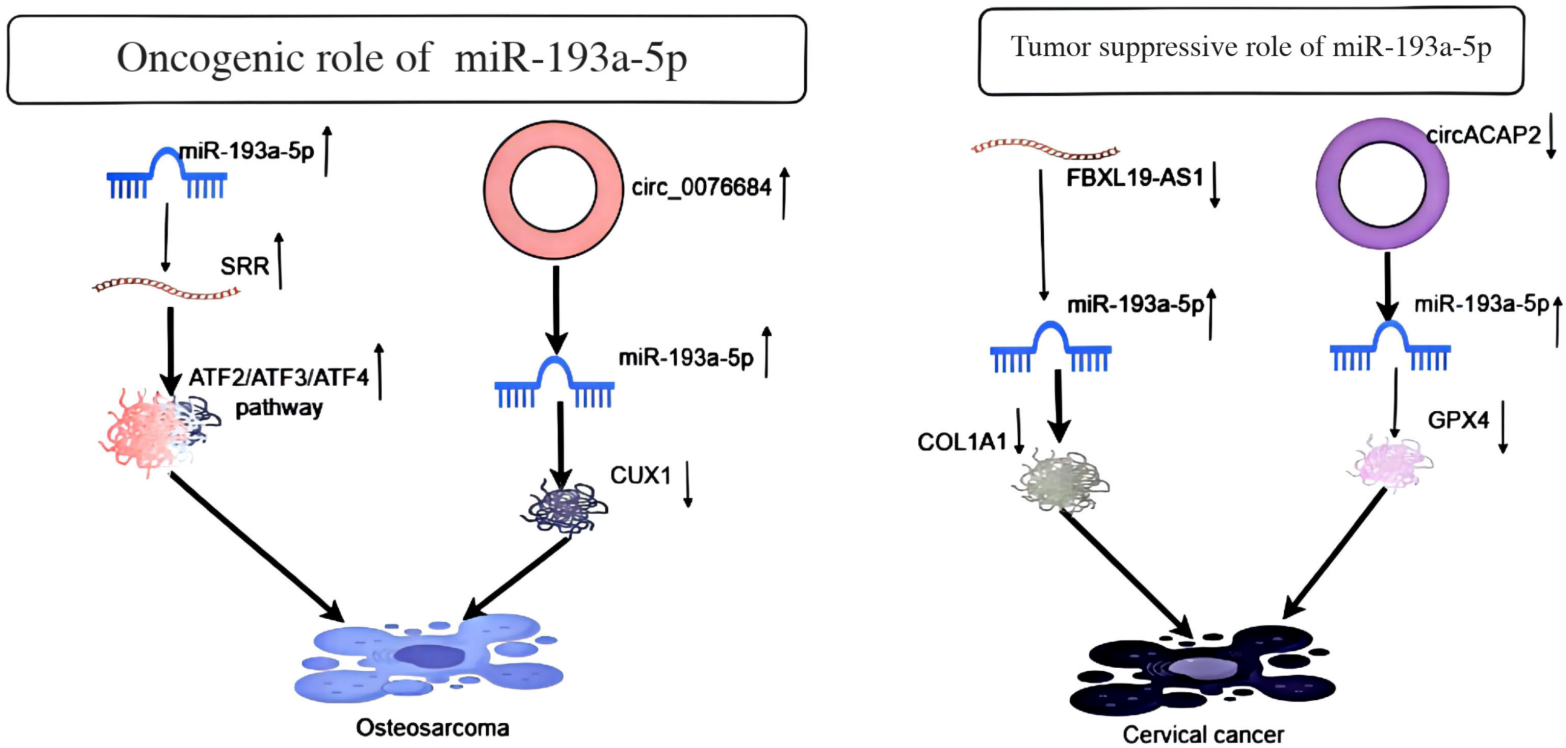
Depicts the oncogenic effect of miR-193a-5p in osteosarcoma and its tumor suppressor role in cervical cancer.

In a study of osteosarcoma cells, sequencing-based miR-omics and quantitative real-time PCR analysis demonstrated that miR-193a-5p is highly expressed in the metastatic osteosarcoma cell line MG63.2 compared to the less metastatic MG63 cell line. This differential expression is associated with DNA methylation in the promoter region. miR-193a-5p inhibits TGF-β, Myc/Max, and ATF2/ATF3/ATF4 signaling pathways, thereby regulating the expression of serine racemase (SRR) and inhibiting the migration and invasion of osteosarcoma (**Figure 5**) (27). Another study revealed that circ_0076684 and RUNX family transcription factor 2 (RUNX2) mRNA are significantly upregulated in osteosarcoma cells due to transcriptional activation mediated by Chromobox homolog 4 (CBX4). The adsorption of circ_0076684 can enhance the expression of homeobox 1 (CUX1) through miR-193a-5p, promoting the proliferation, migration, and invasion of osteosarcoma (28). Lastly, it has been shown that miR-193a-5p is upregulated in osteosarcoma, promoting colony formation, migration, and invasion *in vitro*, as well as metastasis *in vivo*.

IIn a study of cervical cancer cells, FBXL19-AS1 functions as a competitive endogenous RNA (ceRNA) to inhibit the expression of miR-193a-5p (**Figure 5**). miR-193a-5p targets the 3′-UTR site of COL1A1, negatively regulating COL1A1 expression, thereby promoting cell proliferation, migration, invasion, and inhibiting apoptosis (29). Another study revealed that circular RNA circACAP2 inhibits ferroptosis in cervical cancer by targeting miR-193a-5p/GPX4 during malignant progression. As a ceRNA of miR-193a-5p, circACAP2 directly interacts with miR-193a-5p in cervical cancer cells. miR-193a-5p targets GPX4, and circACAP2 promotes GPX4 expression by adsorbing miR-193a-5p in cervical cancer cells. Downregulation of circACAP2 inhibited the viability of cervical cancer cells; however, a miR-193a-5p inhibitor or GPX4 overexpression could reverse these effects. Inhibition of miR-193a-5p or GPX4 overexpression suppressed circACAP2 depletion-induced lipid ROS, iron, and Fe^2+^ in cervical cancer cells (30).

**Figure 6:**
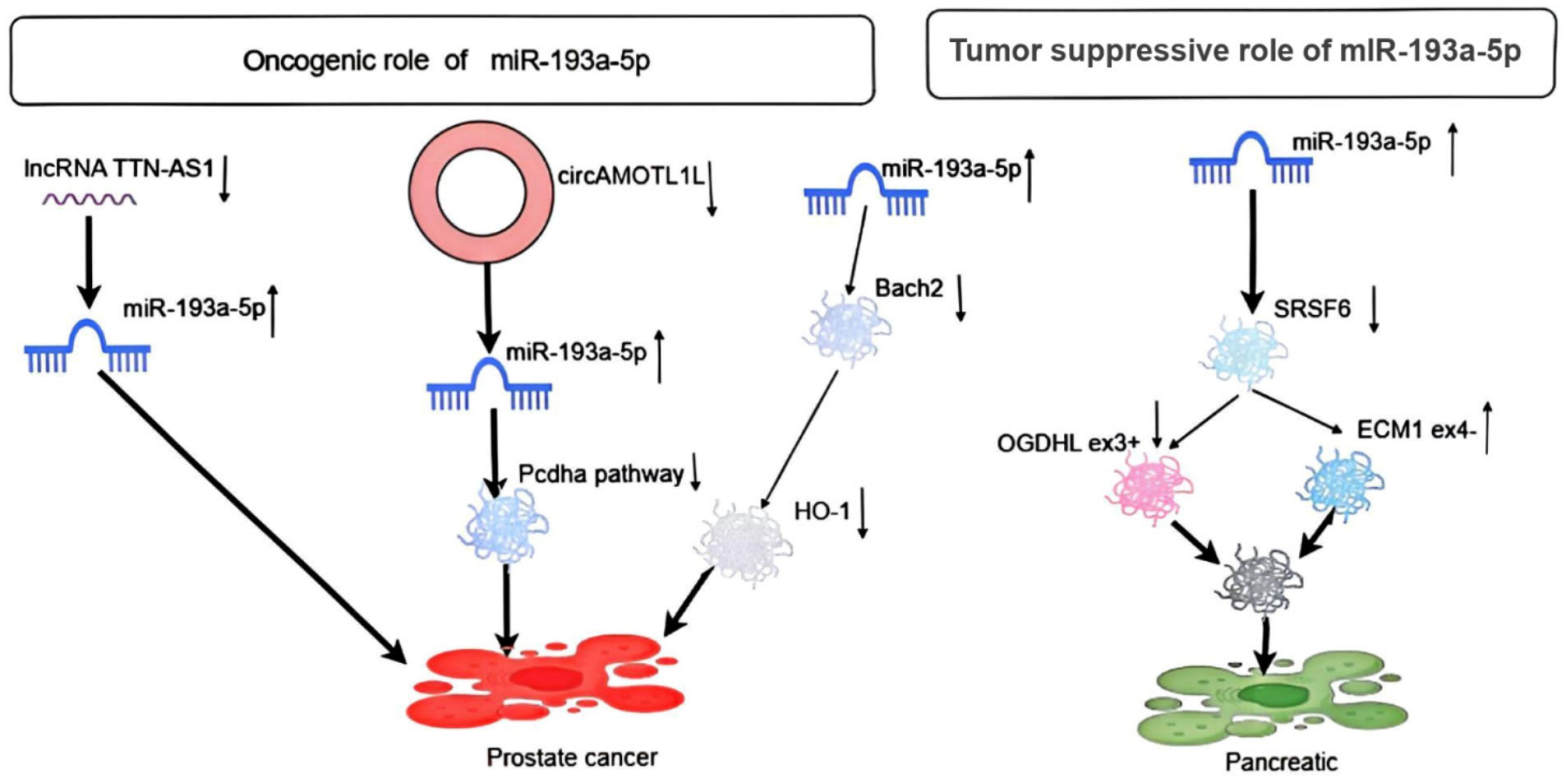
Depicts the oncogenic role of miR-193a-5p in pancreatic and prostate cancer.

In pancreatic cancer cells, miR-193a-5p is upregulated and involved in the activation of serine/arginine-rich splicing factor 6 (SRSF6), OGDHL, extracellular matrix protein 1 (ECM1), and epithelial-mesenchymal transition (EMT) (**Figure 6**) (31). Furthermore, another study showed that miR-193a-5p inhibited pyroptosis and provided protection in pancreatic acinar cells by targeting the circhipk3-mediated NLRP3 pathway and inhibiting the expression of inflammatory signal-related proteins TLR4, MyD88, and NF-κB. Alternatively, miR-193a-5p is a key gene for regulating caerulein-induced AR42J cell damage by targeting tumor necrosis factor receptor-associated factors, and circHIPK3 has been shown to promote pyroptosis in acinar cells by regulating the miR-193a-5p/GSDMD axis (32–34). Finally, luciferase reporter gene assay and RT-qPCR showed that miR-193a-5p is highly expressed in radioresistant pancreatic cancer, potentially enhancing radioresistance by targeting ZFP57 (35).

The regulation of lncRNA TTN-AS1 on miR-193a-5p was verified through RT-PCR assay and Dual-Luciferase reporter assay in prostate cancer cells. The adsorption of lncRNA TTN-AS1 promotes miR-193a-5p, thereby enhancing cell proliferation and inhibiting apoptosis in prostate cancer cells (36). Additionally, another study demonstrated that circAMOTL1L, acting as a sponge, binds miR-193a-5p in PCa cells, alleviating miR-193a-5p’s inhibition of the Pcdha gene cluster (a subset of the cadherin superfamily). Immunofluorescence staining showed that knockdown of miR-193a-5p by its antagonist increased E-cadherin expression and decreased vimentin levels in the cell membrane. Moreover, depletion of miR-193a-5p, combined with circAMOTL1L overexpression, further enhanced E-cadherin expression and reduced vimentin levels. Thus, the dysregulation of the circAMOTL1L-miR-193a-5p-Pcdha8 regulatory pathway, mediated by circAMOTL1L downregulation, contributes to PCa growth *in vivo*. We also show that RBM25 directly binds to circAMOTL1L and induces its biogenesis, while p53 regulates EMT by directly activating the RBM25 gene (37). Another study indicated that miR-193a-5p affects STAT3 and androgen receptor (AR) in prostate cancer (38). Finally, docetaxel-induced upregulation of miR-193a-5p relieves the expression of Bach2, a repressor protein inhibiting the HO-1 gene, by directly targeting the Bach2 mRNA 3′-UTR, thereby partially counteracting docetaxel-induced apoptosis (**Figure 6**) (39).

**Table 1** shows the expression levels of miR-193a-5p in the cell lines.

**Table 1:** Expression of miR-193a-5p or its partners in cell lines.

Tumor type	Targets/regulators and signaling pathways	Cell line	Function	Reference
Papillary thyroid carcinoma	hsa_circ_0001666	TPC-1、IHH-4、GLAG-66	Δ hsa_circ_0001666 (↑↑miR-193a-5p): ↓ Proliferation, migration and invasion	(6)
Ovarian cancer	RBBP6	SKOV3、A2780、HEY、OVCAR3 and Es2	↑↑miR-193a-5p:↓ Proliferation, migration, infestation and↑ apoptosis	(7)
	HOXA7	SKOV3、 OVCAR3、A2780	↑↑ miR-193a-5p:↓ Proliferation, migration, infestation and↑ apoptosis	(8)
Melanoma	LINC01224、NR1D2	A375、MeWo、501Mel	Δ LINC01224 (↑↑miR-193a-5p): ↓ Proliferation, migration, infestation	(9)
	TROY	A375、MeWo	↑↑miR-193a-5p:↓ Proliferation, migration	(50)
Breast cancer	circ_0001667、 Rap2A	Adriamycin (ADM) -resistant breast cancer (BC) cells	Δ circ_0001667 (↑↑miR-193a-5p): ↓ proliferation, migration, and invasion ↓ chemoresistance	(10)
Renal cell carcinoma	TIMP2、HIF1α、VM	786-O、Caki-1、THP-1、HEK 293 T	↑↑miR-193a-5p:↑ proliferation, migration,	(11)
Colorectal cancer	CircRNA_0000392、 PIK3R3、AKT-mTOR	HT29、 HCT116、 SW480、SW837、 SW48、 SW620、RKO	Δ CircRNA_0000392 (↑↑miR-193a-5p): ↓ proliferation, migration, and invasion	(12)
	CUX1、ITSN1	CCC-HIE-2、HCT-8、SW480	↑↑miR-193a-5p:↓ proliferation, migration	(13)
	ERK signal	HT-29、HCT-116 、 SW-480		(14)
	CXCR4	HCT-116 、 SW-480 and HT-29	↑↑miR-193a-5p:↓ proliferation, migration	(15)
	MCM3AP-AS1、SENP1	HCT-8、HCT116、LoVo、HT29 and SW620	Δ MCM3AP-AS1 (↑↑miR-193a-5p): ↓ proliferation, migration, and invasion	(18)
	ZNF746、c-Myc	HCT116、SW480	↑↑miR-193a-5p:↓ proliferation, migration	(19)
Hepatocellular carcinoma	BMF	HepG2	↑↑ miR-193a-5p: ↑ proliferation ↓ apoptosis	(20)
	SPOCK1	HepG2、Hep3b、HL-7702	Δ SPOCK1 (↑↑miR-193a-5p): ↓ Proliferation, infestation	(21)
	CircRNA has_circ_0001806、MMP16	L02、Huh-7、HepG2、SMMC-7721、Bel-7402、HL-7702	ΔCircRNA has_circ_0001806 (↑↑miR-193a-5p): ↓ Proliferation, migration and invasion	(24)
	LncRNA HEIH、 CDK8	huh7、hep3B、smmc-7721、sk-Hep-1、L02	ΔLncRNA HEIH (↑↑miR-193a-5p): ↓Proliferation, migration and invasion	(23)
Gastric cancer	Waveform protein, MMP-9 gene	AGS、MKN-45 and KATO III	↑↑ miR-193a-5p: ↓migration	(25)
	ETS1、CCND1	AGS	↑↑ miR-193a-5p: ↓ Growth, infestation	(26)
Osteosarcoma	SRR、ATF2/ATF3/ATF4 pathway	MG63	↑↑SRR (↑↑miR-193a-5p): ↑ migration	(27)
	circ_0076684、CUX1	MG63、Saos2、143B、 U2OS、hFOB 1.19	↑circ_0076684 (↑↑miR-193a-5p): ↑ Proliferation, migration and invasion	(28)
	NCX2	SaOS-2、U-2OS 、SJSA-1、MG63、hFOB1.19、ATCC	↑↑ miR-193a-5p):↑ Proliferation,migration and invasion, EMT process	(44)
Cervical cancer	FBXL19-AS1、 COL1A1	HUCEC、HeLa、Caski、C-33 A、AV3	ΔFBXL19-AS1 (↑↑miR-193a-5p): ↓ Proliferation, migration and invasion	(29)
	circACAP2、GPX4	SiHa、HeLa	ΔcircACAP2 (↑↑miR-193a-5p): ↓Proliferation, migration and invasion	(30)
Pancreatic	SRSF6、OGDHL、ECM1	Panc-1、 MIApaca-2、 SW1990、BXpc-3、HPDE	Δ SRSF6 (↑↑ miR-193a-5p):↑ Proliferation,migration and invasion, EMT process	(31)
	ZFP57, Wnt/β-catenin pathway	BxPC-3	Δ ZFP57 (↑↑ miR-193a-5p):↑ PCC radioresistance	(35)
Prostate cancer	lncRNA TTN-AS1	DU145、PC3、22RV1、C4-2B 、LNCaP	ΔlncRNA TTN-AS1 (↑↑ miR-193a-5p):↑ Proliferation and ↓ Apoptosis	(36)
	circAMOTL1L、p53、RBM25	PC3、DU145	ΔcircAMOTL1L (↑↑ miR-193a-5p):↑ Proliferation and ↓ Apoptosis	(37)
	Bach2	LNCap、PC3、 DU145、T24、UM-UC-3、WPE-1	↑↑ miR-193a-5p: ↑ Proliferation, migration and invasion	(39)
Rhabdomyosarcoma	SNAIL	RH30、RH41、RD	↑↑ miR-193a-5p:↓ Proliferation, migration and invasion	(40)
Aortic aneurysm	RelB	PCNA、CCND1、CCNE1、CXCR4	↑↑ miR-193a-5p: ↓ Proliferation, migration and invasion	(41)
Non-small cell carcinoma	DLGAP1-AS、DTL	A549、NCI-H1299、NCI-H1975、NCI-H358、SK-MES-1	↑↑ miR-193a-5p: ↓ Proliferation, migration	(45)
	PIK3R3、mTOR、 AKT/mTOR	SPC-A-1sci (high metastatic) and SPC-A-1 (weakly metastatic) cells	↑↑ miR-193a-5p: ↓ Proliferation, migration	(46)
Nasopharyngeal carcinoma	LncRNA LINC01569	HOK、 FaDu、SCC4 、TU212、Hep-2、RAW264.7、THP-1	ΔLncRNA LINC01569 (↑↑ miR-193a-5p):↑Proliferation, migration	(47)
	ERBB2	HET-1A、ESCC Cell line	↑↑ miR-193a-5p: ↓Proliferation, migration	(48)
Adenocarcinoma of the lungs	SNHG17、NETO2	BEAS-2B、A549、H1299、H1650、H1975、CALU-3	↑↑ miR-193a-5p: ↓ Proliferation, migration	(49)
Acute myeloid leukemia (AML)	LncRNA TUG1、Rab10	HL-60、NB4、HS-5	ΔLncRNA TUG1 (↑↑ miR-193a-5p):↓ cell viability ↑apoptosis	(54)
bladder cancer	AP-2α	293T、SV-HUC-1、UM-UC-3	↑↑ miR-193a-5p: ↑ Proliferation, migration ↑Cisplatin resistance	(56)

Δ, knock-down or deletion; RBBP6, RB-binding protein 6; HOXA7, homology frame gene A7; VM, Vasculogenic mimicry in cells; CUX1, CUT-like homology frame 1; ITSN1, intercombinational protein 1; SRR, serine rabbitase; SRSF6, serine/arginine-rich splicing factor; OGDHL, oxoglutarate dehydrogenase-like; ECM1, extracellular matrix protein 1.

↑↑ indicates promotion, ↓ indicates inhibition.

## Animal studies

The tumor-suppressive role of miR-193a-5p has been validated in various animal models of cancer. These studies demonstrate that overexpression or modulation of miR-193a-5p can inhibit cancer progression. For instance, transplantation of cells expressing miR-28-3p and miR-193a-5p in rhabdomyosarcoma models suppressed tumor growth, metastasis, and SNAIL expression, indicating tumor suppressor effects by targeting SNAIL (40). In another study on aortic aneurysms, ReIB inhibition of miR-193a-5p expression exacerbated the proliferation and migration of vascular smooth muscle cells (VSMCs). Additionally, RNA XIST was found to negatively regulate aortic aneurysm cell proliferation by targeting the miR-193a-5p/KLF7 axis (41, 42).

To evaluate the effect of MCM3AP-AS1 modulation of miR-193a-5p on *in vivo* oncogenesis in colorectal carcinoma, Zhou et al. implanted MCM3AP-AS1-silenced LoVo cells into nude mice, demonstrating that MCM3AP-AS1 binds miR-193a-5p, inhibiting its function and significantly promoting tumor proliferation and metastasis (18, 43). *In vivo* experiments in osteosarcoma xenograft models showed that miR-193a-5p targets NCX2; NCX2 knockdown activates AKT by increasing Ca2+, thus promoting epithelial-mesenchymal transition (EMT) and enhancing metastasis to the lung and liver (44). It was demonstrated that miR-193a-5p targets ZFP57, suppressing its expression and upregulating cyclin 1, CDK4, and Bcl-2 to activate the Wnt/β-catenin pathway, enhancing radioresistance in pancreatic cancer cell tumors (35). Silencing of HEIH in a hepatocellular carcinoma xenograft model hindered cell viability, migration, and invasion by regulating the miR-193a-5p/CDK8 axis (23). Silencing lncRNA DLGAP1-AS1 via miR-193a-5p reduced tumorigenesis in NSCLC cells xenografted into nude mice (45). Another study in non-small cell lung cancer showed that miR-193a-5p downregulates the mTOR/PIK3R3 signaling pathway, inhibiting the migration, invasion, and EMT of NSCLC cells (46). Studies in renal cell carcinoma (RCC) xenograft models have shown that inhibition of miR-193a-5p levels in tumor-associated macrophage (TAM) exosomes can inhibit tumor progression and metastasis by inhibiting angiogenic mimicry (VM) and upregulating TIMP2 expression (11). Conversely, in hypopharyngeal carcinoma, downregulation of LINC01569 inhibits macrophage M2 polarization through miR-193a-5p/FADS1 signaling, aiding tumor cells in evading immune surveillance and promoting tumor progression (47). In a xenograft model of oesophageal carcinoma, Liang et al. showed that miR-193a-5p supplementation in KYSE70 cells, subcutaneously injected into immunodeficient mice, inhibited tumor growth by reducing Erb-B2 Receptor Tyrosine Kinase 2 (ERBB2)expression *in vivo* (48).

**Table 2** shows the results of animal studies investigating the effects of miR-193a-5p on tumorigenesis.

**Table 2:** Expression of miR-193a-5p or its partners in Animal models.

Tumor type	Animal models	Results	Reference
Papillary thyroid carcinoma	BALB/c nude mice	Δ hsa_circ_0001666 (↑miR-193a-5p): ↓Tumor size, proliferation	(6)
Renal cell carcinoma	Thymus-free BALB/c nude mice	↑↑miR-193a-5p:↑ Tumor proliferation, migration	(11)
Colorectal cancer	BALB/c nude mice	Δ MCM3AP-AS1 (↑↑miR-193a-5p): ↓ Tumor proliferation, migration	(18)
Hepatocellular carcinoma	BALB/c nu/nu mice	ΔLncRNA HEIH (↑↑miR-193a-5p): ↓ Tumor proliferation, migration	(23)
Osteosarcoma	BALB/c nude mice	Δcirc_0076684 (↑↑miR-193a-5p): ↑Tumor proliferation, migration	(28)
	Male BALB/c nude mice	↑↑ miR-193a-5p):↑ Tumor proliferation, migration invasion, EMT process	(44)
Cervical cancer	Balb/c Nude Mouse	ΔFBXL19-AS1 (↑↑miR-193a-5p): ↓Tumor proliferation, migration	(29)
Pancreatic	Male BALB/c nude mice	Δ SRSF6 (↑↑ miR-193a-5p):↑ Tumor proliferation, migration invasion, EMT process	(31)
	Female BALB/c nude mice	Δ ZFP57 (↑↑ miR-193a-5p):↑ PCC radioresistance	(35)
Prostate cancer	Male BALB/c nude mice	↑↑ miR-193a-5p: ↑ Tumor growth and weight	(39)
Rhabdomyosarcoma	NOD-SCID mice	↑↑ miR-193a-5p:↓ Tumor proliferation, migration	(40)
Nasopharyngeal carcinoma	BALB/c mice	↓LncRNA LINC01569↑miR-193a-5p ↓Tumor growth and weight	(47)
	NOD-SCID mice	↑↑miR-193a-5p:↑ Tumor proliferation, migration	(48)

Δ, knock-down or deletion.

↑↑ indicates promotion, ↓ indicates inhibition.

## Human studies

The downregulation of miR-193a-5p has been validated in clinical specimens from patients with various malignancies. In lung adenocarcinoma, SNHG17, which reduces miR-193a-5p levels, is overexpressed in lung adenocarcinoma tissues and is associated with tumor-node-metastasis stage and poor prognosis (49). In most melanoma cases, miR-193a-3p and miR-193a-5p are downregulated (50). Additionally, GO database analysis revealed that Kirsten rat sarcoma virus oncogene homologs (KRAS), Erb-B2 receptor tyrosine kinase 2 (ERBB2), phosphoinositide-3-kinase regulatory subunit 3 (PIK3R3), mechanistic target of rapamycin kinase (mTOR), myeloid leukemia cell differentiation (MCL1), nucleolin, and spindle-associated protein 1 (NUSAP1) are silenced in miR-193a, implicating their roles in carcinogenesis and cancer progression (12, 51). Furthermore, miR-193a-3p and miR-193a-5p exhibit low expression in endometrial cancer tissues, correlating with adverse clinical parameters (52). One study on endometrial cancer shows that YY1 overexpression directly interacts with miR-193a-5p, promoting cell growth (53).

In leukemic samples, AML patients display significantly reduced miR-193a-5p expression and antileukemic activity of miR-193a-5p, upregulated miR-193a-5p significantly restricted AML cell viability but promoted cell death in AML HL-60 and NB4 cells. Rescue experiments demonstrate that taurine upregulated gene 1 (TUG1) mediates cell viability and death of AML cells by targeting miR-193a-5p (54).

In studies of prostate cancer, osteosarcoma, liver cancer, and pancreatic cancer, miR-193a-5p is upregulated in malignant tumor tissues and positively correlates with tumor-lymph node-metastasis (TNM) stage and N classification. Conversely, miR-193a-5p is downregulated in other malignant tissues, including gastric, cervical, nasopharyngeal, and thyroid cancers. Kaplan-Meier survival analysis from the TCGA database indicates that patients with high miR-193a-5p expression have significantly shorter overall survival times (31). **Table 3** shows the levels of miR-193a-5p and their correlation with clinical outcomes in different cancer types.

**Table 3:** Dysregulation of miR-193a-5p or its chaperones.

Tumor type	Samples	Expression of miR-193a-5p or other genes (tumor vs. normal)	Reference
Papillary thyroid carcinoma	60 patients with PTC	hsa_circ_0001666 downregulation (miR-193a-5p upregulation)	(6)
Ovarian cancer	30 EOC patients	Up-regulation in miR-193a-5p	(7)
Renal cell carcinoma	51ccRCC patients	Up-regulation in miR-193a-5p	(11)
Colorectal cancer	6 pairs of tumor tissues and ANCTs	Downregulation in CircRNA_0000392 (Up-regulation in miR-193a-5p)	(12)
	37 CRC patients and 42 non-cancer patients	Up-regulation in miR-193a-5p	(13)
Hepatocellular carcinoma	50 pairs of tumor tissues and ANCTs	Up-regulation in miR-193a-5p	(20)
	46 pairs of tumor tissues and ANCTs	Downregulation in SPOCK1 (Up-regulation in miR-193a-5p)	(21)
	116 HCC patients	Up-regulation in miR-193a-5p	(22)
	CEO Database: GSE94508, GSE97332	Downregulation in CircRNA has_circ_0001806 (Up-regulation in miR-193a-5p)	(24)
	59 pairs of tumor tissues and ANCTs	Downregulation in LncRNA HEIH (Up-regulation in miR-193a-5p)	(23)
Gastric cancer	TCGA database: 399 tumor tissues and ANCT	Up-regulation in miR-193a-5p	(26)
Pancreatic	40 pairs of tumor tissues and ANCTs	Downregulation in SRSF6 (Up-regulation in miR-193a-5p)	(31)
	30 PC patients	Up-regulation in miR-193a-5p	(35)
Osteosarcoma	25 pairs of tumor tissues and ANCTs	Up-regulation in miR-193a-5p	(44)
Non-small cell carcinoma	48 pairs of tumor tissues and ANCTs	Up-regulation in miR-193a-5p	(45)
Nasopharyngeal carcinoma	6 patients with nasopharyngeal cancer	Up-regulation in miR-193a-5p	(47)
Adenocarcinoma of the lungs	50 pairs of tumor tissues and ANCTs	Up-regulation in miR-193a-5p	(49)
Melanoma	11 patients with Melanoma	Up-regulation in miR-193a-5p	(50)
Acute myeloid leukemia (AML)	23 patients with AML	Downregulation in LncRNA TUG1 (Up-regulation in miR-193a-5p)	(54)

PTC, Thyroid Carcinoma; EOC, Ovarian Cancer; ccRCC, Clear Cell Renal Cell Carcinoma; CRC, Colorectal Cancer; HCT, Hepatocellular Carcinoma; ANCT, Adjacent Non-Carcinogenic Tissue; PC, Pancreatic Cancer; OS, Osteosarcoma; NSCLC, Non-Small Cell Carcinoma; LUAD, Lung Adenocarcinoma.

↑↑ indicates promotion, ↓ indicates inhibition.

## Discussion

MiR-193a-5p is a small RNA molecule that exhibits a dual role in various cancer types, functioning as both an oncogene and a tumor suppressor. In pancreatic, prostate, osteosarcoma, and hepatoma cells, miR-193a-5p is abnormally overexpressed, closely associated with cancer cell proliferation, migration, invasion, and increased resistance to chemotherapeutic agents. Conversely, in other cancer types such as gastric, nasopharyngeal, breast, and cervical cancer, miR-193a-5p acts as a tumor suppressor, often downregulated.

Numerous studies have shown that various long non-coding RNAs (lncRNAs) and circular RNAs (circRNAs) modulate their functions by adsorbing miR-193a-5p or its specific targets. Examples include hsas_circ_0001666, circRNA_0000392, circ_0001667, circ_0001806, circ_0076684, circACAP2, circhipk, circAMOTL1L, LINC01569, TTN-AS1, DLGAP1-AS1, and FBXL19-AS1. These findings highlight the diversity and complexity of miR-193a-5p within the tumor suppressor or oncogenic network. The aberrant upregulation of these lncRNAs and circRNAs is considered a potential mechanism for miR-193a-5p downregulation and genomic variation.

Dysregulation of miR-193a-5p significantly impacts cancer-related signaling pathways. miR-193a-5p interacts with pathways such as extracellular signal-regulated kinase (ERK), TGF-β, Myc/Max, and ATF2/ATF3/ATF4 to regulate its activity. It also interacts with molecules such as KLF7, GSDMD, TLR4, MyD88, and NF-κB to influence inflammation and immune responses. In cell cycle regulation, miR-193a-5p affects cell invasion and proliferation by targeting MMP16 and CDK8. These interactions reveal the complex mechanisms of miR-193a-5p in cancer development.miR-193a-5p inhibits breast cancer progression by targeting SNHG1 and inactivating the oncogene HOXA1. Overexpression of SPOCK1 promotes the proliferation and invasion of HCC cells, but miR-193a-5p suppresses HCC progression by reducing SPOCK1 expression. In colorectal cancer, overexpression of miR-193a-5p inhibits cell migration. The miR-193a-5p/DPEP1 axis regulates hepatoblastoma progression via the PI3K/Akt/mTOR signaling pathway (55). MiR-193a-5p acts as a tumor suppressor in glioma cells by targeting NOVA1 (51). Notably, miR-193a-5p is significantly upregulated in pancreatic cancer, associated with poor prognosis and increased cell migration (49). These examples provide insights for designing cancer-specific therapies. The downregulation of miR-193a-5p or the upregulation of miRNA/circRNA adsorption correlates with the malignant characteristics of cancers such as colorectal cancer, osteosarcoma, prostate cancer, and liver cancer, indicating that miR-193a-5p could serve as a prognostic marker. Low miR-193a-5p expression in poorly differentiated cells prone to metastasis is linked to poor clinical outcomes.

MiR-193a-5p can induce cisplatin resistance by inhibiting AP-2α expression in bladder cancer cells and reduce cisplatin resistance in lung cancer by targeting circ_0048856 (56, 57).miR-193a-5p mediates the development of chemoresistance in tumor cells by post-transcriptionally regulating apoptosis-related genes and signaling pathways, thereby significantly modulating the expression levels of anti-apoptotic proteins such as Mcl-1 and Survivin. In hepatocellular carcinoma (HCC), for example, this miRNA negatively regulates SPOCK1 gene expression, indirectly disrupting the dynamic equilibrium of BCL-2 family proteins and impairing the activation of the p53 signaling pathway, ultimately enhancing tumor cell resistance to apoptotic signals (21). Notably, miR-193a-5p exhibits marked downregulation in the microenvironment of acute myeloid leukemia (AML), suggesting its potential role as an epigenetic regulator in chemoresistance mechanisms (54). Given that the homologous miR-193b-5p has been demonstrated to enhance cisplatin sensitivity in HCC cells by targeting the 3’-UTR of Mcl-1 (58), it is hypothesized that miR-193a-5p may similarly influence Mcl-1 protein stability through analogous epigenetic regulatory patterns, thereby attenuating chemotherapy-induced mitochondria-dependent apoptotic effects. Survivin, as an anti-apoptotic protein, has been shown to promote apoptosis and suppress proliferation in multiple tumor types upon genetic silencing (59). According to research conducted by Jafarlou et al., the concurrent siRNA-mediated suppression of the Mcl-1 and Survivin genes in human monocytic leukemia cells significantly augments their chemosensitivity to chemotherapeutic agents (60). Additionally, Mcl-1-specific siRNA inhibition has been demonstrated to effectively induce apoptosis in leukemia cells and overcome chemoresistance (61). Based on these mechanisms, a precision therapeutic strategy utilizing RNA interference (RNAi) technology is proposed: Bioinformatics-driven screening of key nodes within the miR-193a-5p downstream regulatory network (e.g., Mcl-1/Survivin) could facilitate the design of dual-targeting siRNA molecules. These siRNAs, delivered via lipid nanoparticle or viral vector delivery systems, may achieve synergistic gene silencing. This approach could simultaneously suppress anti-apoptotic protein networks, reactivate tumor cell apoptosis execution programs, and ultimately enhance the efficacy of conventional chemotherapeutic agents while reversing chemoresistance phenotypes.

Many studies emphasize miR-193a-5p’s role in regulating cellular sensitivity to chemical drugs and radiation. Clinically, increasing miR-193a-5p expression is considered a promising strategy to inhibit tumor growth and reduce chemotherapeutic resistance.

miR-193a-5p is a microRNA molecule with remarkable functional plasticity. Its unique “tumor suppressor-oncogenic” dual characteristics make it a critical regulatory node in tumor biology research. This dynamic functional balance stems from the complexity and spatiotemporal specificity of its target gene network. Through interactions with differentially expressed target genes across various tumor types, microenvironments, or disease progression stages, it participates in regulating key malignant phenotypes including cell proliferation, apoptosis, metabolic reprogramming, invasion/metastasis, and therapeutic resistance. At the molecular mechanism level, the dual functionality of miR-193a-5p originates from its dynamic seed sequence matching patterns with the 3’UTR regions of target genes: When acting on oncogenic genes (e.g., cell cycle regulators, anti-apoptotic proteins, or invasion-related enzymes), it exerts tumor-suppressive effects through complete complementary binding and transcriptional repression. Under specific pathological conditions, however, its partial complementary binding to tumor suppressor genes or signaling pathway inhibitors may release constraints on oncogenic pathways, creating pro-tumorigenic effects. This target selectivity shift is influenced by multiple regulatory factors, including RNA-binding proteins (e.g., HNRNP family) modulating miRNA-target complex stability, competing endogenous RNA (ceRNA) networks diluting miRNA effective concentrations, and epigenetic modifications (e.g., promoter methylation or histone acetylation) regulating its spatiotemporal expression. Based on this, we hypothesize that in different tumors, miR-193a-5p may act through mechanisms that interconnect its roles as both tumor-suppressive miRNA and oncogenic miRNA, thereby modulating its functional manifestations. Notably, while this dual functionality has only been confirmed in a few cancers, the phenomenon warrants further investigation. From a signaling network perspective, miR-193a-5p simultaneously interfaces with core oncogenic pathways such as PI3K/AKT/mTOR, Wnt/β-catenin, and TGF-β. Its functional output depends on pathway activation status and combinatorial patterns of downstream effectors—under metabolic stress in the tumor microenvironment, it may enhance cell survival by reprogramming energy metabolism, whereas under genomic instability, it may influence cell fate decisions via DNA damage repair regulation. This dynamic equilibrium often correlates with tumor progression stages: Early-stage tumors may utilize its suppression of proto-oncogenes to maintain genomic stability, while advanced metastatic stages may exploit its regulation of epithelial-mesenchymal transition (EMT)-related factors to promote invasion. In clinical applications, the dual nature of miR-193a-5p presents multidimensional opportunities and challenges for therapeutic strategy design. As a therapeutic target, its functional plasticity necessitates precise molecular subtyping systems: Single-cell sequencing could decode its expression profiles across tumor subpopulations, while AI algorithms could predict functional status correlations with drug sensitivity, enabling differentiated intervention strategies. Therapeutic development could involve lipid-encapsulated miRNA mimics to restore tumor-suppressive functions or chemically modified antisense oligonucleotides (ASOs)/small-molecule inhibitors to block oncogenic activity, requiring tissue-specific nanocarrier delivery systems to avoid off-target effects. Notably, miR-193a-5p’s interaction with the tumor immune microenvironment reveals novel therapeutic dimensions: Its regulation of immune checkpoint molecules (e.g., PD-L1) may influence T-cell infiltration (62), while exosome-mediated intercellular communication could reshape immunosuppressive microenvironments (63), providing rationale for combination immunotherapy. However, translational challenges include its dose-dependent bidirectional effects—low concentrations may induce compensatory pathway activation through partial target inhibition, while high concentrations risk normal tissue toxicity from overregulation. This demands mathematical model-based dosing systems for dynamic optimization. Additionally, tumor escape mechanisms involving miRNA sponge adsorption or extracellular vesicle-mediated therapeutic miRNA depletion may require combinatorial targeting with gene editing technologies.

Although miRNA sequence-based nucleic acid therapies show promise, issues related to safety and efficacy must be addressed before clinical application. These include the stability and delivery efficiency of miRNA mimics, potential immune responses, and long-term safety profiles. Once these challenges are overcome, miR-193a-5p-based treatments could become effective cancer therapies offering hope to patients.
